# Isoniazid‐induced pellagra in a patient with Crohn's disease

**DOI:** 10.1002/ccr3.2740

**Published:** 2020-02-19

**Authors:** Hela Kchir, Haythem Yacoub, Hajer Hassine, Nadia Maamouri

**Affiliations:** ^1^ Gastroenterology B Department La Rabta Hospital Tunis Tunisia; ^2^ Faculty of Medicine of Tunis El Manar University Tunis Tunisia

**Keywords:** Crohn's disease, isoniazid, niacin, pellagra

## Abstract

Isoniazid preventive therapy in malnourished patients with Crohn's disease has a potential of inducing pellagra but still a very rare situation. No cases of isoniazid‐induced pellagra in patients with Crohn's disease were reported in the literature. Pellagra can be easily treated if timely diagnosed.

## Graphical abstract

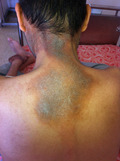

## 1 INTRODUCTION

Pellagra is a multinutrient deficiency disease caused by a deficiency of niacin and/or tryptophan. It is a nutritional disorder characterized by suggestive clinical manifestations: dermatitis, diarrhea, and dementia (“the 3 Ds”). Pellagra may be secondary to other pathological conditions such as pheochromocytoma and the use of certain drugs such as isoniazid.

Pellagra is a multinutrient deficiency disease caused by a deficiency of niacin and/or its precursor, tryptophan, and often involving another vitamin B especially vitamin B6 deficiency. It is a nutritional disorder characterized by suggestive clinical manifestations such as dermatitis, diarrhea, and dementia (“the 3 Ds”). Severe forms of pellagra can lead to death (the 4th D).^1^ Administration of isoniazid (INH) to latent tuberculosis patients to prevent evolution to active disease is commonly indicated in patients under immunosuppressive therapy such as antitumor necrosis factor‐alpha, and it is known as INH preventive therapy.^2^ Symptoms related to pellagra are often observed in malnourished people but may be a complication of other pathological conditions such pheochromocytoma and the use of certain drugs: isoniazid, 6‐mercaptopurine, 5‐fluorouracil, and chloramphenicol. Isoniazid may precipitate pellagra, particularly in malnourished people.^3^ We report a case of isoniazid‐induced pellagra in a patient with Crohn's disease (CD).

## 2 CASE REPORT

**Figure 1:**
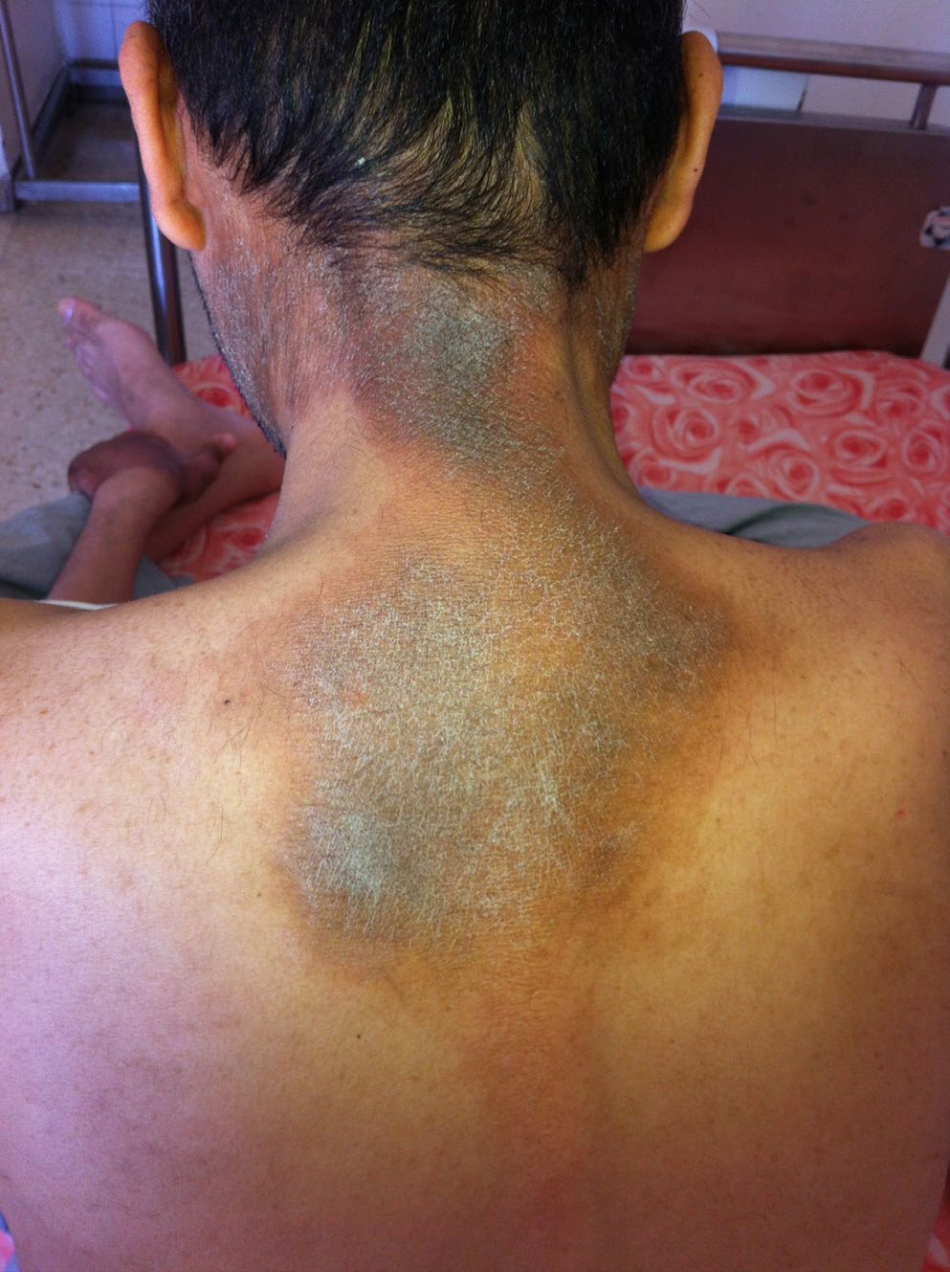
Well‐demarcated and slightly pigmented erythematous plaques at the back of the neck and upper back of a patient with Crohn's disease treated with INH

**Figure 2:**
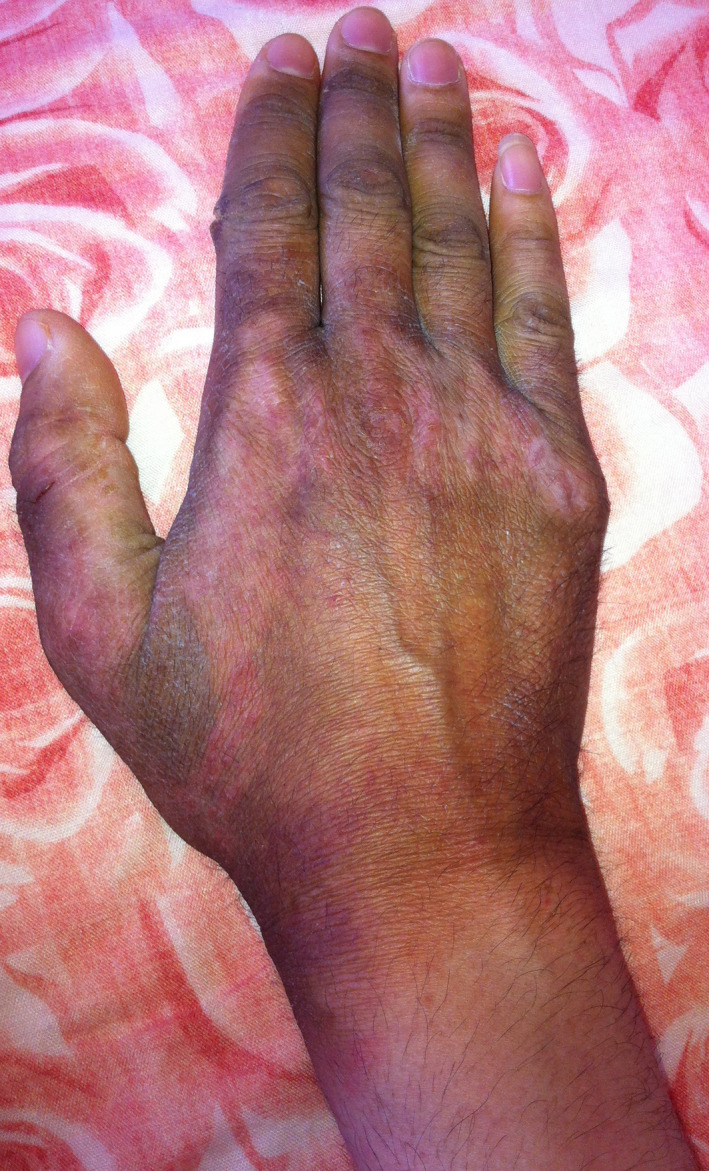
Sunburn‐like lesions of the hand

**Figure 3:**
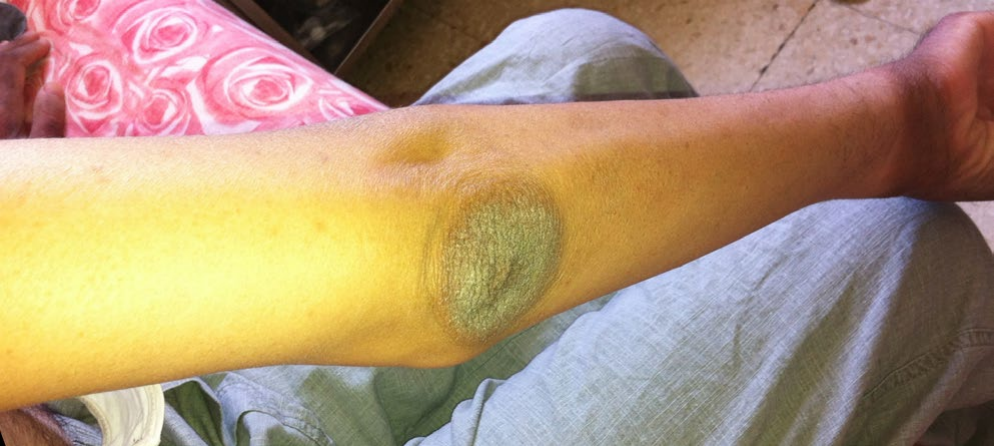
Hyperkeratotic and well‐demarcated plaques on the right elbow

A 31‐year‐old man with no history of alcoholism or cigarette smoking was diagnosed with ileal and extensive colonic Crohn's disease in 2001 associated with primary sclerosing cholangitis. The patient was treated for years with oral mesalamine and prednisolone. In 2010, the patient was seen at our center for a new severe relapse of CD. The treatment was changed to azathioprine due to the inadequate response to prednisolone. The disease frequently relapsed despite azathioprine therapy. These findings led in 2014 to the decision to start adalimumab (antitumor necrosis factor‐α therapy). Before initiating any biologic therapy, screening for the infectious disease was performed. The patient underwent a chest X‐ray, screening for tubercle bacilli test in sputum and urine, hepatitis B and C viral infections, human immunodeficiency virus (HIV), and Epstein‐Barr virus (EBV). The results were negative. The QuantiFERON^®^‐TB Gold test (QFT‐G), which is a blood test helping in the detection of mycobacterium tuberculosis, was positive. INH preventive therapy was started orally at 3 mg/kg/d (recommended dose after therapeutic drug monitoring). Fourteen weeks later, our patient developed erythematous plaques with slight pigmentation on the upper part of the neck and the back with clear zone of demarcation between the affected and normal skin (Figure 1), resembling sunburn erythema on the dorsum of his hands (Figure 2), and well‐demarcated hyperkeratotic plaques on the elbows (Figure 3). At that time, he had frequent diarrheal stools (14/d) that are bloody at times. He also had anorexia and difficulty in oral alimentation. On interrogation, episodes of delirium and behavioral change in the form of crying and laughing out loud that was markedly different from his usual behavior were noted. At physical examination, his weight was 57 Kg, height was 1.82 m, and body mass index (BMI) was 17.2 Kg/m^2^. Based on these findings, pellagra was suspected. The laboratory findings including hemoglobin, platelet counts, liver function tests, thyroid hormones, renal function tests, and serum electrolytes were normal. Serum niacin level was very low (6.4 μmol/L: normal range, 38‐58 μmol/L). Pharmacovigilance survey confirmed INH‐induced pellagra. The management plan included INH withdrawal, and nutritional review and vitamin supplementation. The patient was treated with niacin (200 mg/d) orally. Three weeks later, the rash disappeared and diarrhea stopped. The patient was started on adalimumab 160 mg on the first injection, 80 mg in the second injection, and then 40 mg every 2 weeks. After the third injection of adalimumab, clinical symptoms improved.

## 3 DISCUSSION

Pellagra is a multinutrient deficiency disease involving vitamin B deficiency especially niacin and/or its precursor and tryptophan, and it is clinically characterized by four Ds: diarrhea, dermatitis, dementia, and death. Pellagra was first described by Gaspar Casal in poor populations in 1735.^4^ This condition can be due to dietary deficiency of niacin or vitamin B3, and then, it is called primary pellagra. Pellagra may also occur as a result of relatively rare conditions affecting tryptophan or niacin metabolism such as alcoholism and severe infections. It also may be a side effect of certain drugs that inhibit tryptophan metabolism.^4^ Patients, who are on poor diets and on long‐term use of isoniazid, may manifest clinical signs of deficiency. INH inhibits the intestinal absorption of niacin. The alteration of the metabolism of INH in predisposed patients may lead to its accumulation as an analogue of niacin, inhibiting its endogenous production and leading to pellagra.^5^ In this case, we speculate that INH is a direct causative factor for pellagra. Our patient's nutrition is also poor in terms of frequency and diversity. CD is associated with decreased absorption of niacin and seems to have a role in developing this nutritional disorder. A review of the literature confirms the paucity of reports of pellagra as a complication of CD.^6^, ^7^, ^8^, ^9^, ^10^ The three “Ds” of pellagra were observed in our patient, and this association is present in only 22% of patients.^11^ This case demonstrates the classical case of pellagra, in a patient on INH preventive therapy. No cases of isoniazid‐induced pellagra in patients with CD were reported in the literature. In pellagra, coenzyme nicotinamide adenine dinucleotide (NAD) and NAD phosphate (NADP) levels are low due to niacin deficiency. NAD and NADP participate in numerous processes including amino acid metabolism and the generation of high‐energy phosphate bonds. This would explain why pellagra mainly affects fast‐renewal tissues such as skin and the digestive tract, and tissues with high‐energy needs, such as the brain neurons.^7^

Skin lesions usually involve friction and sun‐exposed areas like the face, neck, and feet. They are usually bilateral and symmetrical; there is usually a clear demarcation of the lesions from the normal skin. Skin lesions appear like sunburn or more commonly hyperpigmented and desquamated plaques in sun‐exposed sites.^5^, ^12^ The diagnosis should rely on the specific skin lesions with their characteristic zone of demarcation and typical distribution.^5^ In other dermatitis associated with CD, there is no clear area of demarcation or symmetrical distribution of lesions. Neuropsychiatric manifestations of pellagra can manifest as nervousness, insomnia, hallucinations, headaches, irritability, lack of concentration, and anxiety.^13^ Episodes of delirium and behavioral change in the form of crying and laughing out loud were noted in our patient.

The diagnosis of pellagra should rely primarily on specific clinical findings. It should be noted that niacin assays are reliable tests but not necessary for the diagnosis of pellagra.^5^ The administration of niacin has a good impact on pellagra. Treatment should continue for at least 4 weeks, and the daily recommended dose is 300 mg of niacin.^5^ The oral dose is then reduced to 50 mg every 8‐12 hours until recovery is complete. Treatment should also include other B vitamins, zinc, magnesium, and a high‐calorie diet.^4^ In the case of isoniazid‐induced pellagra, discontinuation of the causative drug is indicated.^14^ In our patient, favorable clinical course after vitamin supplementation and INH withdrawal was observed.

## 4 CONCLUSION

The administration of isoniazid to latent tuberculosis patients is commonly indicated in patients with CD under immunosuppressive therapy. Since pellagra still occurs, we should take this disease into account in cases of malnourished patients with CD, especially with serious diarrhea, also for those treated with immunomodulators and under isoniazid preventive therapy. Pellagra can be easily treated if timely diagnosed.

## CONFLICT OF INTEREST

None.

## AUTHOR CONTRIBUTIONS

Hela Kchir, Haythem Yacoub, and Hajer Hassine designed the concept of the manuscript and involved in the definition of intellectual content. Hela Kchir and Haythem Yacoub involved in the literature search. Hela Kchir, Haythem Yacoub, Hajer Hassine, and Nadia Maamouri involved in the manuscript preparation. Hela Kchir acted as Guarantor for the research.

## PATIENT'S CONSENT

Yes.
